# Embedment of Biosynthesised Silver Nanoparticles in PolyNIPAAm/Chitosan Hydrogel for Development of Proactive Smart Textiles

**DOI:** 10.3390/nano15010010

**Published:** 2024-12-25

**Authors:** Dominika Glažar, Danaja Štular, Ivan Jerman, Barbara Simončič, Brigita Tomšič

**Affiliations:** 1 Department of Textiles, Faculty of Natural Sciences and Engineering, University of Ljubljana, Aškerčeva 12, 1000 Ljubljana, Slovenia; dominika.glazar@ntf.uni-lj.si (D.G.); barbara.simoncic@ntf.uni-lj.si (B.S.); 2 National Institute of Chemistry, Hajdrihova 19, 1000 Ljubljana, Slovenia; danaja.stular@gmail.com (D.Š.); ivan.jerman@ki.si (I.J.)

**Keywords:** smart textiles, responsive hydrogel, poly(N-isopropylakrylamide)/chitosan, green biosynthesis, silver nanoparticles, antibacterial activity, UV protection

## Abstract

A smart viscose fabric with temperature and pH responsiveness and proactive antibacterial and UV protection was developed. PNCS (poly-(N-isopropylakrylamide)/chitosan) hydrogel was used as the carrier of silver nanoparticles (Ag NPs), synthesised in an environmentally friendly manner using AgNO_3_ and a sumac leaf extract. PNCS hydrogel and Ag NPs were applied to the viscose fabric by either in situ synthesis of Ag NPs on the surface of viscose fibres previously modified with PNCS hydrogel, or by the direct immobilisation of Ag NPs by the dehydration/hydration of the PNCS hydrogel with the nanodispersion of Ag NPs in the sumac leaf extract and subsequent application to the viscose fibres. Compared to the pre-functionalised PNCS application method, the in situ functionalisation imparted much higher concentration of Ag NPs on the fibres, colouring the samples brown to brown-green. These samples showed more than 90% reduction in the test bacteria *E. coli* and *S. aureus* and provided excellent UV protection. In this case, the PNCS hydrogel acted as a reservoir for Ag NPs, whose release was based on a diffusion-controlled mechanism. Despite the Ag NPs decreasing the responsiveness of the PNCS hydrogel, the moisture management was still preserved in the modified samples. Accordingly, the PNCS hydrogel is a suitable carrier for biosynthesized Ag NPs to tailor the protective smart surface of viscose fibres.

## 1. Introduction

Textiles serve as a highly conducive medium for the proliferation and excessive growth of microorganisms. The presence of body fluids and environmental moisture, optimal temperatures, and an abundance of nutrients in the form of contaminants—such as food residues, dead skin cells, bodily secretions (sweat, urine, faeces), blood, and others—create ideal conditions for the rapid and uncontrolled microbial growth on textile surfaces. These contaminated textiles can act as vectors for the transmission of potentially pathogenic microorganisms, posing a significant risk for cross-infection between users [1,2,3]. Of particular concern is the potential for cross-contamination between different environments, such as between the workplace and the home, which could facilitate the emergence and spread of novel diseases.

Considering these risks, the antimicrobial functionalization of textiles through the application of antimicrobial agents is of critical importance. This treatment not only inhibits the growth of microbes responsible for unpleasant odours, thus contributing to the maintenance of personal hygiene, but more importantly provides an effective protection against potentially pathogenic microorganisms [4,5,6]. Silver nanoparticles (Ag NPs) are among the most widely used antimicrobial agents for textile treatment due to their broad-spectrum antimicrobial activity [6,7,8,9,10,11,12,13]. Namely, in the controlled release mechanisms, Ag^+^ ions and Ag NPs released from a material can interact with the negatively charged cell membranes of microorganisms [11,12,13,14,15,16,17,18,19,20,21]. Due to their nanoscale size, Ag NPs are also able to penetrate bacterial cells, where they can bind to thiol groups in enzymes or nucleic acids, which disrupts important physiological processes and ultimately leads to the death of the bacterial cells. In addition, both Ag^+^ ions and Ag-NPs can generate reactive oxygen species (ROS) in the presence of oxygen through photocatalysis. Although ROS are natural byproducts of cellular respiration, their excessive accumulation can lead to oxidative damage and cell death. Finally, Ag NPs can also interfere with cellular signal transduction, disrupting the biochemical pathways essential for microbial cell activity and growth.

The use of substances that pose risks to human health and the environment during nanoparticle synthesis, along with the formation of toxic byproducts, is one of the major challenges in the antimicrobial functionalization of textiles using Ag NPs. A significant advancement is the adoption of biosynthesis methods for Ag NPs, which utilise natural plant extracts as reducing and stabilising agents [21,22,23,24,25,26,27,28], whereby employing plant extracts with known medicinal properties is doubly beneficial, as they can provide complementary bioactive effects and further improve the antimicrobial efficacy of Ag NPs. Accordingly, sumac (genus: *Rhus*) has already shown its advantageous phytochemicals and reducing properties in biosynthesis of Ag NPs [29,30,31,32,33,34,35,36]. It is known for its high concentration of bioactive compounds, including tannins, flavonoids, and phenolic acids. These compounds exhibit strong reducing and stabilising capabilities, making sumac ideal for biosynthesis of metallic nanoparticles. Sumac leaves, specifically, are rich in tannins, which are potent antioxidants. These tannins can act as reducing agents, facilitating the reduction of silver ions (Ag^+^) to metallic silver (Ag^0^) and capping the particles to stabilise them, which helps prevent agglomeration and enhances the stability of the nanoparticles. Recent studies have already demonstrated the efficacy of sumac extracts in synthesising Ag NPs with favourable properties like small size, stability, and high antimicrobial activity, supporting the potential of sumac-based Ag NPs in the textile field [37,38,39,40]. Accordingly, in our previous study incorporating sumac-synthesised Ag NPs onto fabrics, we showed promise for durable antimicrobial textiles, with applications in healthcare, sportswear, and everyday clothing, where controlling odour and maintaining hygiene are beneficial [39,40].

Stimuli-responsive hydrogels represent a new generation of highly cross-linked and hydrated polymers for the surface modification of textiles. They offer exceptional potential for thermoregulation and moisture management to increase wearer comfort. These polymer networks can respond dynamically to external stimuli (e.g., changes in temperature, pH, light, ionic strength, etc.) that trigger the uptake and release of water from the polymer network, causing the hydrogel particles to swell or de-swell. When applied to textile fibres, this behaviour influences the porosity and breathability of the modified textiles and allows the fabrics to proactively adapt to changing conditions by effectively regulating moisture and maintaining a balanced microclimate on the skin [41,42,43,44,45,46,47,48,49]. In this context, temperature- and pH-responsive polymers based on poly(N-isopropylacrylamide) and chitosan (PNCS hydrogel) are among the most extensively studied [47,48,49,50,51,52,53,54,55,56]. The PNCS hydrogel swells at temperatures below the lower critical solution temperature (LCST) of poly(N-isopropylacrylamide), which is 32 °C, and at a pH below the pKa of chitosan, which is 6.5, while it de-swells at higher temperatures and pH levels. In addition, PNCS hydrogel can be chemically modified to incorporate bioactive components that further improve the performance and health benefits of the textiles by allowing precise control over when and how much of the bioactive substances are released [54,55,56]. In our previous studies, the PNCS hydrogel thus showed the potential to develop non-cytotoxic smart textile materials that can release AgCl nanocrystals that function according to the principle of controlled release, whereas by synthesising PNCS hydrogels with incorporated β-cyclodextrins and increased chitosan concentration, a dual antimicrobial effect was achieved, characterised by the controlled release of the antimicrobial savoury essential oil and the simultaneous formation of a bio-barrier by chitosan [57,58].

While biosynthesized Ag nanoparticles (Ag NPs) have been explored individually in various applications, the integration of these nanoparticles with a dual pH- and temperature-responsive hydrogel represents a significant advance in the development of smart textiles that combine moisture management for increased comfort and adaptability to fulfil modern demands for high-performance, wearable materials with additional antibacterial and UV-protective activity. The aim of this study was therefore to use a stimuli-responsive PNCS hydrogel as a carrier for Ag NPs, biosynthesised using a sumac leaf extract as a natural reducing and stabilising agent, and to incorporate this composite into viscose fibres for the production of smart textiles. The viscose fabric was chosen for its softness and breathability, which are desirable for smart textiles, although it traditionally lacks moisture management and antibacterial properties. Special focus was giving to optimising the incorporation of Ag NPs into PNCS hydrogel particles to achieve improved stability and reactivity. Accordingly, two innovative methods were exploited. The first method involved in situ functionalisation, in which the biosynthesis of the Ag NPs takes place in the presence of a viscose fabric coated with PNCS hydrogel, whereby the nanoparticles are integrated directly into the fabric. In the second method, a pre-functionalisation, the Ag NPs were embedded in the PNCS hydrogel by dehydrating and rehydrating them in a nanodispersion of Ag NPs and the sumac extract before the pre-functionalised PNCS hydrogel was applied to the viscose fibres. With this novel combination, we aimed to drive the development of next-generation responsive finishes for smart textiles that combine moisture management for comfort with added protective properties. In particular, our goal was to gain new insights into the responsive mechanisms of the functionalized PNCS hydrogel and its interaction with biosynthesized Ag NPs, as well as to investigate how the application parameters influence the chemical, morphological, and functional performance of such textile composites.

## 2. Materials and Methods

### 2.1. Materials

Throughout the experiments, a 100% viscose fabric with a weight of 110 g/m^2^ (warp density: 44 threads/cm; weft density: 34 threads/cm) was used. Silver nitrate (AgNO_3_), an N-isopropylacrylamide monomer (NiPAAM), cross-linker N,N-methylenebisacrylamide (MBA), and initiator ammonium persulfate were purchased from Sigma Aldrich. Chitosan (DD = 95%, η = 159 mPa∙s) was purchased from Chitoclear, Primex, Iceland. A reactive organic–inorganic sol–gel precursor iSys HPX (CTH, Döhlau, Germany) was used. Sumac leaves (*Rhus typhina* L.) were collected in Istria, Croatia, in springtime. Before the experiment, the leaves were air-dried at room temperature.

### 2.2. Synthesis of the Poly-NiPAAm/Chitosan (PNCS) Hydrogel

The poly-NiPAAm/chitosan (PNCS) hydrogel was prepared using the surfactant-free dispersion co-polymerisation method described by Lee et al. [59]. First, a 0.3% chitosan–water solution was prepared containing 1 g chitosan and 3 mL glacial acetic acid in 300 mL distilled water, which was stirred overnight at room temperature. Subsequently, the chitosan solution was degassed with nitrogen for 30 min, and then 7 g of NiPAAm and 0.21 g of MBA were added with vigorous stirring (320 rpm). The temperature of the reaction medium was raised to 50 °C, and after 40 min, 0.9 g APS was added to initiate the NiPAAm/chitosan co-polymerisation. The reaction medium became milky after 5 min, and the reaction was carried out for 3 h at 50 °C under a nitrogen atmosphere. To remove the unreacted monomer and oligomeric impurities, the reaction solution was dialysed (4 Spectra/Por, Fisher Scientific, Waltham, MA, USA) against deionised water for at least 1 week. The hydrogel produced from poly-NiPAAm and chitosan (PNCS) had a concentration of 23.7 g/L.

### 2.3. Preparation of Sumac Leaves’ Extract

Dried sumac leaves were crushed by hand and added to distilled water at a concentration of 20 g/L. The mixture was boiled for 20 min, then cooled to room temperature, and finally filtered through a sieve.

### 2.4. Functionalisation of Viscose Fabric

A polysiloxane matrix was first applied to the viscose samples to increase the adsorption of the Ag NP-functionalised PNCS hydrogel on fibres. For this purpose, the reactive organic–inorganic sol–gel precursor iSys HPX was mixed with distilled water at a concentration of 15 g/L to promote the hydrolysis of the precursor. The viscose fabric was treated with the hydrolysed precursor using the pad-dry-cure method, with 100 ± 2% wet pick-up, drying at 100 °C for 90 s, and curing at 150 °C for 5 min.

The samples thus prepared were then modified using two methods, namely, via the application of the PNCS hydrogel with subsequent in situ synthesis of Ag NPs and via the direct application of a functionalised PNCS hydrogel with previously embedded Ag NPs.

The first method of functionalisation was carried out as follows. The PNCS hydrogel was applied to viscose fabric samples using a pad-dry method, including full immersion in the PNCS hydrogel dispersion following drying under the same conditions previously described. After the application of the PNCS hydrogel, the in situ synthesis of Ag NPs was carried out using the infrared dyeing apparatus Starlet-2 DL-6000 Plus (DaeLim Starlet Co., Ltd., Seoul, Republic of Korea). Individual samples of 8 g in mass were placed in containers filled with 200 mL of the AgNO_3_ solution of two concentrations, 1.0 and 5.0 mM. The samples were then treated at a temperature of 60 °C for 10 min with constant stirring. After 10 min, another 200 mL of aqueous sumac leaves’ extract was added to the containers, obtaining a final fabric-to-liquor ratio of 1:50. The samples were then treated for 1 h at 60 °C with constant stirring. The bath was then cooled to 20 °C, and the samples were treated at this temperature for another 30 min. By lowering the temperature, we aimed to trigger the swelling of the PNCS hydrogel particles by simultaneously absorbing synthesised Ag NPs into the PNCS hydrogel particles. At the end of the treatment, the samples were squeezed out on a two-roll padder and air-dried.

In the second functionalisation method, the PNCS hydrogel particles were pre-functionalised via the introduction of Ag NPs. First, the Ag NPs were synthesised. A solution of AgNO_3_ and the sumac leaf extract was poured into the container, maintaining a volume ratio of 1:1 between the AgNO_3_ solution and the sumac extract. The Ag NPs were then synthesised for 1 h at a temperature of 60 °C with constant stirring. The synthesis of Ag NPs was carried out for both concentrations of the AgNO_3_ solution, namely, 1.0 mM and 5.0 mM. After the synthesis was completed, the Ag NP dispersion was cooled to 20 °C. Meanwhile, the PNCS hydrogel particles were dehydrated by heating the PNCS hydrogel dispersion to 60 °C, causing the hydrogel particles to shrink and release bound water from their structure. The released water was carefully removed and replaced with an equal amount of AgNO_3_ dispersion. The mixture was slowly stirred at room temperature for 30 min to allow the PNCS hydrogel particles to swell gradually and absorb the Ag NP dispersion. The PNCS hydrogel with embedded Ag NPs was stored in a refrigerator for 24 h to ensure that the hydrogel swelled completely. After 24 h, the functionalised PNCS hydrogel with Ag NPs was applied to the viscose fabric samples using the pad-dry method under the same conditions described above, whereas after padding, the samples were left to air-dry.

**Figure 1:**
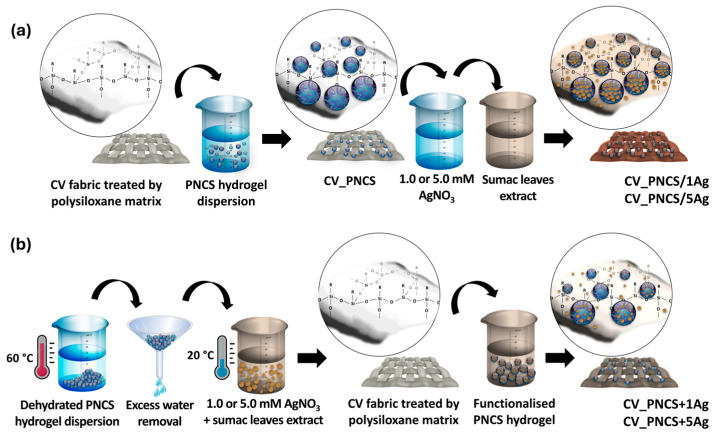
A schematic presentation of viscose fabric modification with PNCS hydrogel functionalised via in situ synthesis of Ag NPs (**a**) and via the direct application of a functionalised PNCS hydrogel with previously embedded Ag NPs (**b**).

For comparison, the PNCS hydrogel alone was applied to the viscose samples. Moreover, the viscose samples were also modified with the in situ-synthesised Ag NPs without the prior application of the PNCS hydrogel. The corresponding sample codes with descriptions of their functionalisation are presented in Table 1. A schematic presentation of the functionalisation procedure is shown in Figure 1.

### 2.5. Analysis and Measurements

#### 2.5.1. Field Emission Scanning Electron Microscopy (FE-SEM) and Energy-Dispersive Field Emission Scanning Electron Microscopy (EDS)

To investigate the surface morphology and elemental composition of the PNCS hydrogel and Ag NP particles on the surface of viscose fibres, FE-SEM and EDS analyses were performed using a Thermo Fisher Scientific Quattro S scanning electron microscope equipped with an energy-dispersive X-ray spectrometer (EDS) from Oxford Instruments (Ultim Max 65, Abingdon, UK). A 1-kilovolt voltage was used to capture the element spectra, generate elemental maps, and obtain backscatter images. Before the analysis, a thin carbon coating was applied to the fabric surface.

#### 2.5.2. Inductively Coupled Plasma-Mass Spectroscopy (ICP-MS)

The concentration of silver on the functionalised samples was determined by ICP-MS using a PerkinElmer SCIED Elan DRC spectrophotometer (Waltham, MA, USA). A sample of 0.5 g was prepared in a Milestone microwave system by acid decomposition with 65% HNO_3_ and 30% H_2_O_2_. Silver concentrations are given as the mean values of two measurements made for each sample.

In addition, an ICP-MS analysis was performed to investigate the Ag release behaviour of the functionalised samples. For this purpose, 1 g of each sample was immersed in 10 mL of a physiological saline solution and placed in a shaker at 37 °C for 8 and 24 h. After the indicated immersion times, the samples were carefully blotted between two filter papers to remove excess liquid and then air-dried. The amount of Ag released from the sample was determined as the difference in silver concentration on the functionalized samples before and after the experiment.

#### 2.5.3. Fourier Transform Infrared (FT-IR) Spectroscopy

FT-IR spectra were obtained from a Spectrum 3 FT-IR spectrometer (PerkinElmer, Inc., Waltham, MA, USA) equipped with an ATR diamond crystal cell with a refractive index of 2.0. The spectra were recorded over the range 4000–600 cm^−1^, with a resolution of 4 cm^−1^, and averaged over 16 spectra. For the purpose of the analysis, the FT-IR spectra of the studied samples were normalised over the recorded range, i.e., 4000–600 cm^−1^.

#### 2.5.4. Colour Measurements

The absorption and reflection of the untreated and functionalised viscose samples were recorded in a wavelength range of 300–800 nm using a Varian Cary 1E UV/Vis spectrophotometer (Varian, Sidney, NSW, Australia) with a DRA-CA-301 integration sphere and Solar Screen software 5.3. From the reflection (R) values of the samples obtained at different wavelengths, the colour strength (K/S) was calculated as follows [60]:

(1) KS=1−R22R,

The colour co-ordinates of the studied samples were determined using a Datacolour Spectraflesh 600 PLUS-CT spectrophotometer (Trenton, NJ, USA). The measurements were performed using a 9 mm aperture under D65 illumination and a 10◦ observation angle. Ten measurements of CIELAB colour co-ordinates were provided for each studied sample, and the colour difference (ΔE*) was calculated using the following equation [60]:

(2) $$\Delta E^{*}=\sqrt{{\left(\Delta L^{*}\right)}^{2}+{\left(\Delta a^{*}\right)}^{2}+{\left(\Delta b^{*}\right)}^{2}},$$

where ΔL*, Δa*, and Δb* are differences between the lightness (L*), green–red (a*), and blue–yellow (b*) colour co-ordinates of the two samples.

#### 2.5.5. Moisture Content

The moisture content was measured thermogravimetrically using a moisture analyser (MS-70 Moisture Analyser equipped with WinCT–Moisture software 3.00, A&D, Tokyo, Japan). The samples were preconditioned at 65% relative humidity at 20 and 40 °C for 24 h. Afterwards, they were dried at 105 °C. During the drying process, the instrument measured the mass of the sample in 2 s intervals and displayed the reduction in moisture. The final moisture content (MC) was calculated as follows:

(3) $$MC=\frac{m_{o}-m_{f}}{m_{o}}\times 100\;\left(\%\right),$$

where m_o_ is the initial mass of the sample (g), and m_f_ is the final mass of the completely dried sample (g). MC is reported as the mean value of three measurements.

From the MC results, the swelling degree triggered by temperature change (*S_T_*) was determined as follows:

(4) $$S_{T}=\frac{MC_{S}-MC_{D}}{MC_{D}}\times 100\;\left(\%\right),$$

where MC_S_ is the MC of the functionalised viscose sample determined at 20 °C when the poly-NiPAAm in the PNCS hydrogel is in a swollen, hydrated phase, and MC_D_ is the MC-functionalised viscose sample determined at 40 °C when the poly-NiPAAm in the PNCS hydrogel is in a de-swollen, dehydrated phase.

#### 2.5.6. Water Uptake

To evaluate the pH responsiveness of the functionalised PNCS hydrogel, the viscose samples of known mass were immersed in buffer solutions of different pH, i.e., 3 and 8, for 30 min at a temperature of 20 °C. Afterwards, the samples were taken from the buffers and weighed. Water uptake (WU) was determined using the following equation:

(5) $$WU=\left(\frac{m_{w}-m_{0}}{m_{0}}\right)\times 100\;\left(\%\right),$$

where m_w_ is the weight of the sample taking up water [g], and m_0_ is the initial weight of the sample [g]. WU is reported as the mean value of five measurements.

From the WU results, the swelling degree triggered by pH change (S_pH_) was determined as follows:

(6) $$S_{pH}=\frac{WU_{S}-WU_{D}}{WU_{D}}\times 100\;\left(\%\right),$$

where WU_S_ is the WU of the functionalised viscose sample determined at 20 °C and pH 3 when the chitosan in the PNCS hydrogel is in a swollen, hydrated phase, and WU_D_ is the WU of the functionalised viscose sample determined at 20 °C and pH 8 when the chitosan in the PNCS hydrogel is in a de-swollen, dehydrated phase.

#### 2.5.7. Antibacterial Activity

Bacterial reduction on the functionalised viscose samples was determined for the Gram-positive bacteria *Staphylococcus aureus* (*S. aureus*; ATCC 6538) and the Gram-negative bacteria *Escherichia coli* (*E. coli*; ATCC 25922) using the ASTM E2149-01 [61] standard method. A fabric sample of 1.0 g was immersed in 20 mL of a bacterial suspension of 10^5^ CFU/mL in a flask, which was then shaken using a wrist-action shaker for 1 h. Afterwards, 40 μL of each suspension was spread on nutrient agar and incubated at 37 °C for 24 h. For each sample, two parallels were performed, and each parallel was spread on four agar plates, giving eight counts per sample in total. The reduction in bacterial growth, *R*, was calculated as follows:

(7) $$R=\frac{B-A}{B}\times 100\;\left(\%\right),$$

where B is the number of bacterial colony-forming units (CFUs) recovered from the inoculated, untreated control sample swatches in the flask at an incubation time of 24 h, and A is the number of bacteria recovered from the inoculated functionalised test sample swatches in the flask at an incubation time of 24 h.

In addition, the size of the inhibition zone was determined using the ISO 20645 Agar diffusion plate test [62]. For this purpose, the tested circular-cut samples were placed on an agar plate inoculated with the bacterium *E. coli* and incubated at 20° or 37 °C for 48 h to evaluate the temperature-dependent release of the biosynthesised Ag NPs from the PNCS hydrogel. The size of the inhibition zone was calculated as follows:

(8) $$H=\frac{D-d}{2},$$

where H is the inhibition zone in mm, D is the total diameter of the viscose sample and the inhibition zone in mm, and d is the diameter of the sample in mm. All tests were carried out in duplicate.

#### 2.5.8. UV-Protective Properties

The UV-protective properties of the studied viscose samples were determined according to the AATCC TM 183 standard [63] by measuring the transmittance of the samples with a Lambda 850+ UV/Vis spectrophotometer (Perkin Elmer, Beaconsfield, UK). Three transmittance measurements were performed in the wavelength range of 290–400 nm at different angles of the warp orientation of the samples. To determine the UV protection factor (UPF), the average transmission value at wavelengths of 315–400 nm (UV-A) and 290–315 (UV-B) was calculated using the following equation:

(9) $$UPF=\frac{\sum_{\lambda =280}^{400}E_{\lambda }\times S_{\lambda }\times \Delta \lambda }{\sum_{\lambda =280}^{400}E_{\lambda }\times S_{\lambda }\times T_{\lambda }\times \Delta \lambda },$$

where *λ* is the wavelength, E_λ_ is the relative erythermal spectral effectiveness, S_λ_ is the solar spectral irradiance, T_λ_ is the spectral transmittance of the specimen, and ∆λ is the measured wavelength interval in nm.

The UPF values were used to determine the UPF rating and protection categories according to the guidelines of the Australian/New Zealand Standard for Sun-Protective Clothing—Evaluation and Classification (AS/NZS, 2017) [64]. UPF values ranging from 15 to 30 indicate UPF ratings in the “minimum” protection category. UPF values from 30 to 50 correspond to “good” protection, while UPF values of 50 and above (50+) are considered to provide “excellent” protection.

## 3. Results and Discussion

### 3.1. Morphological and Chemical Properties of the Modified Viscose Samples

**Figure 2:**
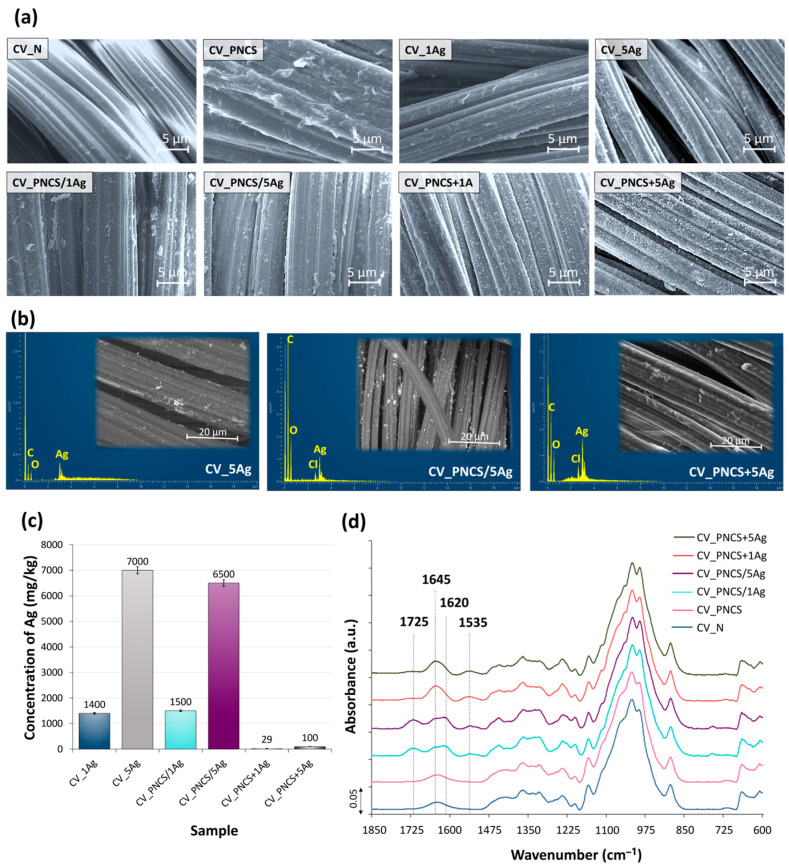
(**a**) SEM images of untreated and modified samples at 3 K magnification; (**b**) EDS spectra of modified CV_5Ag, CV_PNCS/5Ag, and CV_PNCS + 5Ag samples, with corresponding SEM/BSE images as insets; (**c**) Ag concentration in the analysed samples; (**d**) IR-ATR spectra of the analysed samples.

The morphological properties of the modified viscose samples were examined using SEM (Figure 2a). Both the application of the PNCS hydrogel and the in situ synthesis of the Ag NPs increased the roughness of the smooth surface of the viscose fibres. On the surface of the CV_PNCS sample, the particles of the PNCS hydrogel are clearly visible in the form of oval protrusions about 0.6 μm in size. It can also be concluded from this that the PNCS hydrogel particles are not evenly distributed over the surface of the viscose fibres but are rather concentrated at the contact surfaces between the fibres. The SEM images of the CV_1Ag and CV_5Ag samples show the presence of spherical-shaped Ag NPs, the concentration of which on the fibre surface of the CV_5Ag sample is much higher than that of the CV_1Ag sample. This was to be expected due to the higher concentration of AgNO_3_.

Regardless of the functionalisation of the PNCS hydrogel, PNCS particles in the form of oval protrusions can be observed on the surface of the CV_PNCS/xAg and CV_PNCS + xAg samples, indicating that the functionalisation with Ag NPs did not affect the morphology of the PNCS hydrogel. On the contrary, the functionalisation process had a major impact on the concentration of PNCS hydrogel particles on the surface. In fact, the application of pre-functionalised PNCS in one step led to a much higher deposition of hydrogel than the two-step application with Ag NPs synthesised in situ. Therefore, it can be assumed that in the CV_PNCS/xAg samples, a certain amount of PNCS hydrogel particles was desorbed from the surface of the viscose fibres into the finishing bath of the sumac leaf extract during in situ synthesis of Ag NPs.

A reliable differentiation between PNCS hydrogel particles and Ag NPs cannot be made based on the SEM images. Therefore, an SEM/BSE and EDS analysis of the particles was performed considering only the viscose fabric samples treated with the highest concentration of AgNO_3_ during Ag NP synthesis (samples CV_5Ag, CV_PNCS/5Ag, and CV_PNCS + 5Ag). The SEM/BSE images of the samples (inserts in Figure 2b) showed small, bright particles on the surface of the viscose fibres, which are spherical and unevenly distributed over the sample surfaces. Agglomerates or clusters are also visible in some areas. The EDS analysis (Figure 2b) of the bright particles on the fibre surface of the investigated samples confirmed the presence of Ag and, thus, the formation of Ag NPs, as the EDS spectra clearly show a peak at 2.984 keV, which is characteristic of Ag.

Unfortunately, we could not determine the particle size due to the thermoplastic properties of the viscose fibres. Therefore, SEM/BSE imaging could only be performed at a lower magnification of 2000× because, at a higher magnification of 10,000×, which would allow a particle size analysis, the samples started to “slip”, resulting in blurred images. However, based on the scale and a previous study investigating the influence of sumac leaf extract concentration on the green synthesis of Ag NPs [40], we can say with certainty that the average particle size was around 100 nm, while the clusters were >1 µm in size.

The concentration of Ag NPs in the samples was determined by the ICP-MS analysis (Figure 2c), which revealed a much higher Ag concentration on the surface of the PNCS hydrogel-modified viscose samples with in situ-synthesised Ag NPs compared to the samples modified with a pre-functionalised PNCS hydrogel. Accordingly, the CV_PNCS/xAg samples showed a comparable Ag concentration to the CV_1Ag and CV_5Ag samples without the PNCS hydrogel but a 52- and 65-times higher Ag concentration than the corresponding CV_PNCS + xAg samples, respectively. This confirms that the functionalisation process had a significant impact on the concentration of Ag NPs formed on the samples. In the case of the CV_PNCS/xAg samples, the contact of the CV fibres with the AgNO_3_ solution and the sumac leaf extract in the finishing bath during the in situ synthesis of the Ag NPs allowed not only for the absorption of the Ag NPs into the PNCS hydrogel but also their adsorption on the surface of the CV fibres. This was not the case for the CV_PNCS + xAg samples, in which PNCS with the embedded Ag NPs was applied to the CV sample.

The application of a PNCS-functionalised hydrogel affected the chemical changes in the viscose fabric. A comparison of the IR-ATR spectra of the modified samples with the spectrum of the untreated sample (CV_N) in Figure 2d shows the formation of two new absorption bands at 1645 and 1535 cm^−1^, corresponding to the stretching vibrations of the C=O group of amide I and the NH group vibrations of amide II [65]. Amide I confirms the presence of poly-NiPAAm in the hydrogel, while amide II indicates the presence of both poly-NiPAAm and chitosan. In addition to the amide I and II bands, two new absorption bands at 1620 and 1725 cm^−1^ were observed in the IR-ATR spectra of the two CV_PNCS/xAg samples. This band is likely due to carbonyl groups (C=O) originating from compounds in the sumac extract used during the synthesis process, which might have absorbed into the viscose fibres. This result is consistent with previous studies that have identified similar absorption bands between 1700 and 1720 cm^−1^ due to carbonyl stretching vibrations in phytochemicals from plant extracts [33,66]. These carbonyl groups are known to contribute to the reduction and stabilisation of AgNPs on modified textiles, supporting the role of the sumac extract as a reducing and capping agent in Ag NP formation. The presence of this absorption band undoubtedly proves the active involvement of the sumac extract in Ag NP synthesis on the viscose substrate and underpins its dual function in the green synthesis process. Contrarily, these absorption bands were not observed in the spectra of the samples impregnated with the pre-functionalised PNCS hydrogel, consistent with the low Ag concentration determined for both CV_PNCS + xAg samples.

### 3.2. Assessment of Colour Change

**Figure 3:**
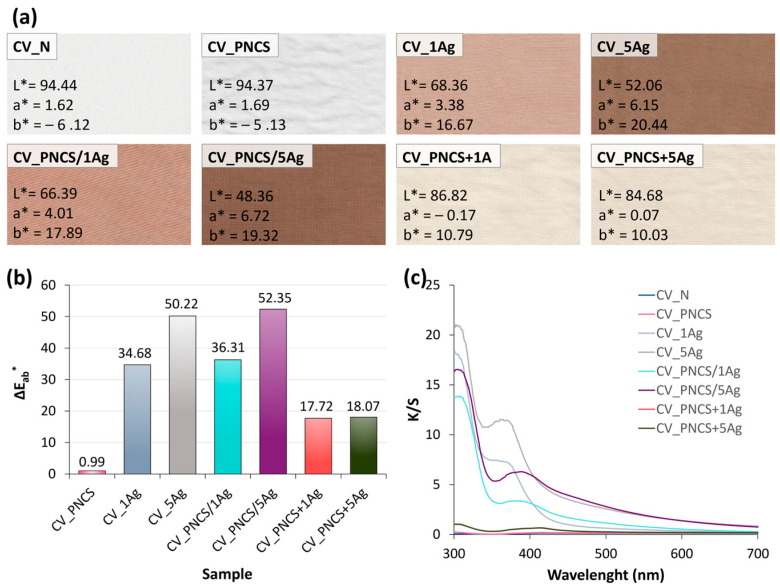
(**a**) Photo images of the untreated and studied modified samples with corresponding CIE L*a*b* values; (**b**) colour change (ΔE_ab_*) of the studied modified samples; (**c**) colour strength (K/S) spectra of the untreated and studied modified samples.

The in situ biosynthesis of Ag NPs using the sumac extract resulted in the viscose fabric taking on a brownish to brownish-green hue, with increasing concentrations of Ag NPs producing a darker colour (Figure 3a). The CIE L*a*b* colour co-ordinates of the CV_xAg samples show that the L* value decreased and the a* and b* values increased, indicating that the CV_5Ag sample became darker and slightly more reddish and yellowish compared to CV_1Ag, respectively (Figure 3a). Accordingly, higher colour change ΔE_ab_* was determined for the CV_5Ag sample and CV_1Ag sample compared to the untreated CV_UN sample (Figure 3b). The same trend is observed for the CV_PNCS/xAg samples, although the decrease in brightness with increasing Ag NP concentration in the presence of the PNCS hydrogel was more pronounced than for the CV_xAg samples. In addition, the increase in a* and b* co-ordinates was also slightly more pronounced, which, in turn, reflected the slightly higher ΔE_ab_* values of both CV_PNCS/xAg samples compared to the CV_xAg versions.

In contrast to the in situ functionalisation of the PNCS hydrogel, the application of the pre-functionalised PNCS hydrogel did not lead to a significant colour change. Both CV_PNCS + xAg samples were significantly lighter and only slightly yellowish after modification. This lighter colour and, thus, low ΔE_ab_* values can undoubtedly be explained by the much lower concentration of Ag NPs incorporated into the hydrogel structure during the dehydration and subsequent rehydration of the PNCS hydrogel particles in the presence of the Ag NP dispersion in the sumac extract. In addition, the CV_PNCS + xAg samples were in contact with the finishing bath for a much shorter time during application, which also resulted in less adsorption of the pre-functionalised PNCS hydrogel particles on the surface of the viscose fibres than in the CV_PNCS/xAg samples.

The colour strength spectra (K/S values) of the CV_xAg and CV_PNCS/xAg samples (Figure 3c) show a prominent peak at 360 nm for the CV_xAg sample, which shifts slightly to 388 nm for the CV_PNCS/xAg sample. Both observed peaks agree well with the surface plasmon resonance (SPR) absorption peak characteristic of Ag NPs [33,39,40,66,67]. The observed red shift in the CV_PNCS/xAg sample could suggest the aggregation of Ag NPs, possibly due to the concentrating in the PNCS hydrogel particles, as it is known that a shorter wavelength of the SPR peak indicates the smaller-size nanoparticles [67]. Accordingly, this interaction likely affected the optical properties and thus implies the presence of clustered nanoparticles in the hydrogel.

### 3.3. Functional Properties

#### 3.3.1. Temperature and pH Responsiveness of the Functionalised PNCS Hydrogel

**Figure 4:**
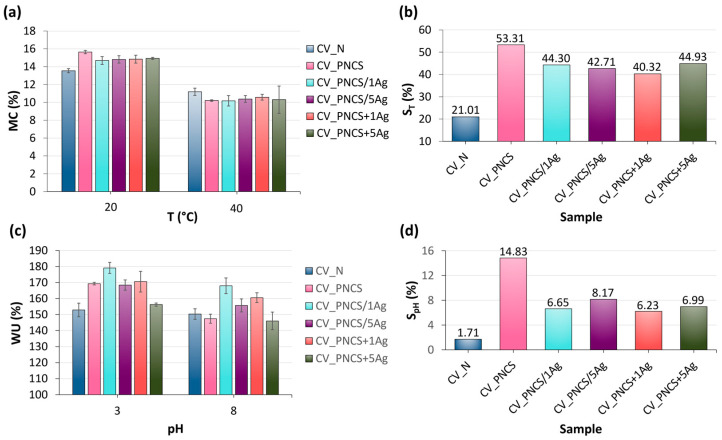
(**a**) The moisture content (MC) of the untreated and modified samples, and (**b**) the swelling ratio of the PNCS hydrogel triggered by temperature change (S_T_); (**c**) the water uptake (WU) of the untreated and modified samples with the (**d**) swelling ratio of the PNCS hydrogel triggered by pH change (S_pH_).

The influence of biosynthesised Ag NPs on the swelling/de-swelling of the PNCS triggered by changes in temperature or pH of the immediate environment was investigated and the results are presented in Figure 4. The swelling capacity of the hydrogel is largely determined by its degree of cross-linking, which directly effects its ability to absorb and retain water. This capacity is usually quantified as the ratio between the mass of the hydrogel in the hydrated state and its mass in the fully dehydrated state [68]. Accordingly, the effect of Ag NPs on the temperature sensitivity of the PNCS hydrogel was investigated by measuring the moisture content (MC) at temperatures of 20 °C and 40 °C, which are below and above the LCST (32 °C) of the temperature-responsive poly-NiPAAm in the PNCS hydrogel (Figure 4a). It can be seen that the samples have a higher MC at 20 °C than at 40 °C, which is due to the hydrophilicity of poly-NiPAAm at temperatures below the LCST. Accordingly, the hydrogel-treated samples (CV_PNCS, CV_PNCS/xAg and CV_PNCS + xAg) showed higher MC values compared to CV_N, as the PNCS hydrogel was able to swell and absorb moisture from the environment. Figure 4a also shows that the presence of Ag NPs slightly influenced the swelling of the PNCS hydrogel, as the MC values for the CV_PNCS/xAg and CV_PNCS + xAg samples were slightly lower compared to CV_PNCS. At 40 °C, the CV_N sample exhibited the highest MC value, while the de-swelling of the PNCS hydrogel in the modified samples led to a decrease in MC values due to the released moisture. The presence of Ag NPs did not significantly affect the de-swelling activity of the PNCS hydrogel, as the CV_PNCS/xAg and CV_PNCS + xAg samples exhibited very similar MC values at 40 °C compared to the CV_PNCS sample.

To gain a more detailed insight into the effects of the presence of Ag NPs and the functionalisation process on the temperature responsiveness of the PNCS hydrogel, the swelling ratio triggered by a change in temperature, S_T_, was further determined (Figure 4b). The presence of Ag in the CV_PNCS/xAg and CV_PNCS + xAg samples resulted in approximately 10% lower S_T_ values compared to the CV_PNCS sample, which can be attributed to their lower swelling capacity at 20 °C. These results are consistent with previous studies [57]. However, the S_T_ values of the CV_PNCS/xAg and CV_PNCS + xAg samples were about two-times higher than that of the unmodified CV_N sample. The comparison of the swelling ratios of the samples modified with the in situ-functionalised and the pre-functionalised PNCS hydrogel shows that it cannot be said with certainty that the functionalisation process of the PNCS hydrogel with biosynthesised Ag NPs influenced the temperature responsiveness of the PNCS hydrogel, as the S_T_ values fluctuate randomly.

The influence of Ag NPs on the pH responsiveness of the PNCS hydrogel due to the presence of chitosan was investigated by measuring the water uptake (WU) at pH 3 and pH 8 at 20 °C (Figure 4c). This pH-dependent behaviour modulates the hydrophilic or hydrophobic nature of the PNCS hydrogel. As expected, the sample exhibited higher WU values in acidic conditions compared to alkaline. This can be attributed to the protonation of the chitosan amino groups at pH values below 6.5 and their deprotonation at pH values above 6.5. Similarly, the CV_PNCS/xAg and CV_PNCS + xAg samples also showed increased WU in acidic media and reduced WU in alkaline media.

Analogous to the results of the moisture content (MC), the pH-induced swelling ratio (S_pH_) was derived from the WU values (Figure 4d). The considerably lower S_pH_ values compared to the S_T_ values are reasonable, as the WU experiments were performed at 20 °C, a temperature at which the PNCS hydrogel partially swells due to the hydrophilic nature of the poly-NiPAAm in the hydrogel. It is also evident that the presence of biosynthesised Ag NPs led to a reduction in the swelling ratio by approximately 52% compared to the CV_PNCS sample. However, the S_pH_ values of the CV_PNCS/xAg and CV_PNCS + xAg samples were 3.6- to 4.8-times higher than that of the unmodified CV_N samples, confirming the presence of pH responsiveness of the modified samples. Similarly to the temperature responsiveness, the functionalisation process had no effect on the inherent pH sensitivity of the PNCS hydrogel.

#### 3.3.2. Antibacterial Activity and Stimuli-Controlled Release of Biosynthesised Ag

**Figure 5:**
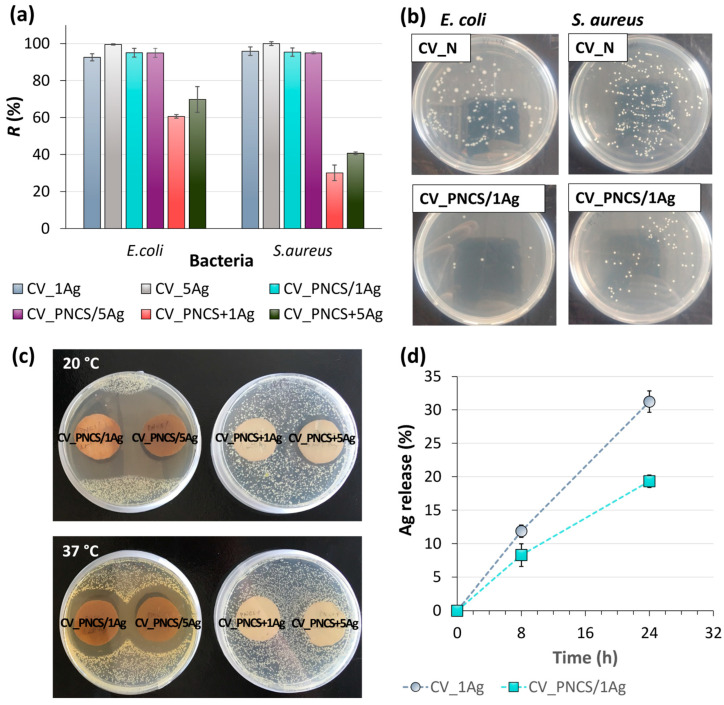
(**a**) The growth reduction of bacteria *E. coli* and *S. aureus* in contact with the studied samples; (**b**) studied bacteria colonies grown on the agar plates after being in contact with the CV_N and CV_PNCS/1Ag samples; (**c**) the inhibition zone formed around the studied CV_PNCS/xAg and CV_PNCS + xAG samples after incubation at 20 and 37 °C; (**d**) silver (Ag) release from the CV_1Ag and CV_PNCS/1Ag samples.

Undoubtedly, the presence of Ag NPs influenced the antibacterial activity of the modified samples analysed. The antibacterial activity of Ag is directly influenced by its concentration on the surface of textile fibres. As shown in Figure 5a,b, the results of the growth reduction of Gram-negative *E. coli* bacteria and Gram-positive *S. aureus* bacteria are expected, as they are well consistent with the Ag concentration determined via the ICP-MS analysis (Figure 2c). Accordingly, the Ag concentration in the CV_PNCS + xAg samples was only sufficient to achieve adequate antibacterial activity against *E. coli*, while the growth reduction of *S. aureus* was insufficient. The reason for this is the higher efficacy of Ag against Gram-negative bacteria, which is related to the concentration of peptidoglycans that act as a defence against Ag in the cell walls of both types of bacteria but are more abundant in the cell walls of Gram-positive bacteria. In contrast, the CV_xAg and CV_PNCS/xAg samples showed excellent antibacterial activity against both tested bacteria, as, in these cases, the in situ biosynthesis of Ag guaranteed a sufficiently high Ag concentration to achieve excellent antibacterial activity.

In continuation, to assess the temperature-responsive release of biosynthesised Ag NPs from the PNCS hydrogel, the size of the inhibition zone was measured. A suspension of *E. coli* bacteria was spread on agar plates, and the samples were placed on these plates and incubated at 20 °C and 37 °C for a certain time. The results are shown in Figure 5c. It is noticeable that the inhibition zones were larger after incubation at 20 °C than at 37 °C. Regardless of the Ag concentration, a comparable zone of inhibition of about 1.8 cm was formed for both the CV_PNCS/1Ag and CV_PNCS/5Ag samples at 20 °C, whereas, at 37 °C, a zone of inhibition of about 0.9 cm was observed for both samples. This result was unexpected, as previous studies [57,58] showed the opposite behaviour, where the controlled release of active substances from the PNCS hydrogel occurred at temperatures above the LCST due to the transition from a swollen to a de-swollen phase. Thus, the active substance was released from the PNCS hydrogel at temperatures above the LCST of poly-NiPAAm and pH conditions above the pKa of chitosan, as the hydrogel desorbed the active substance or squeezed it out into the environment. The observed opposite behaviour of the release of the biosynthesised Ag NPs from the PNCS hydrogel in the swollen phase can be explained by a diffusion-controlled mechanism [69]. This mechanism is influenced by the porosity of the hydrogel and the size of the active substance molecules/particles. In the swollen state, the hydrogel provides a medium that facilitates the movement of the active substance. In this case, the release is driven by a concentration gradient, where the concentration of the active substance inside the hydrogel is higher than in the surrounding medium. Consequently, the active substance diffuses from areas of high concentration (inside the hydrogel) to areas of lower concentration (outside the hydrogel). Therefore, the small size of the biosynthesised Ag NPs allowed them to diffuse through the porous structure of the swollen PNCS hydrogel at 20 °C. However, in the collapsed, de-swollen state at 37 °C, the porous structure of the hydrogel contracted due to the cross-linked polymer chains, which restricted the diffusion of the Ag NPs from the hydrogel into the environment. On the other hand, in the case of the CV_PNCS + xAg samples, no inhibition zone was detected for the CV_PNCS + 1Ag sample; an inhibition zone of about 0.4 cm was observed for the CV_PNCS + 5Ag sample after incubation at 20 °C but was again barely detectable at 37 °C. Undoubtedly, the latter can be ascribed to the low concentration of Ag achieved via the pre-functionalisation of the PNCS hydrogel. Nonetheless, following the process of the functionalisation of the PNCS hydrogel via the in situ biosynthesis of Ag NPs, the PNCS hydrogel can serve as a reservoir for the biosynthesised Ag NPs and enable their release at temperatures below the LCST of poly-NiPAAm.

In continuation of the research, the influence of the PNCS hydrogel on the Ag release behaviour from CV fibres was determined. Accordingly, samples CV_1Ag and CV_PNCS/1Ag were immersed in a physiological saline solution and the amount of Ag released was determined as a function of immersion time, i.e., after 8 and 24 h. The results presented in Figure 5d show that Ag release from the CV_1Ag sample increased with prolonged immersion time, leading to a reduction in Ag content of 12% and 31% after 8 and 24 h, respectively. In contrast, the presence of the PNCS hydrogel significantly reduced Ag release, with only an 8% and 19% decrease in Ag content observed for the CV_PNCS/1Ag sample over the same time periods. While a slight burst of Ag release was observed in the first 8 h, the release rate subsequently stabilised and gradually increased over the following 16 h of immersion. These results undoubtedly demonstrate the diffusion-controlled release mechanism of Ag from the surface of the CV_PNCS/1Ag sample and support the long-term potential of PNCS hydrogel as a Ag NP carrier, allowing their controlled release into the medium to effectively inhibit microbial growth.

As textiles are worn or used in direct contact with the skin, the cytotoxicity of antibacterial fibres is an important issue. The results of Ag release show that the Ag concentration released from the CV_PNCS/1Ag samples exceeded the cytotoxic threshold for Ag-NP reported in the literature over the time periods studied, i.e., 124 and 290 ppm after 8 and 24 h, respectively. Namely, Ag NP concentrations of 50 ppm or more have been shown to cause significant DNA damage [70]. The cytotoxicity of Ag NPs is indeed primarily influenced by their concentration and exposure time, but also by the properties of the nanoparticles. Spherical Ag NPs with a size of about 100 nm are generally considered less cytotoxic compared to smaller nanoparticles [71,72]. In a similar study in which viscose fibres were decorated with Ag nanoparticles synthesised via a green in situ route using a guava leaf extract as a reducing and stabilising agent, fibres with comparable Ag concentrations to the CV_1Ag and CV_PNCS/1Ag samples exhibited only marginal cytotoxicity [73]. Notably, normal human epidermal keratinocytes and dermal fibroblasts retained greater than 80% viability after 48 h of exposure, indicating low toxicity and good biocompatibility. Since the PNCS hydrogel has been reported to be non-toxic (as shown in our previous study) [74], further studies are needed to evaluate the cytotoxicity of the PNCS/xAg-modified samples and confirm their safety for skin contact applications.

#### 3.3.3. UV Protection Activity

**Figure 6:**
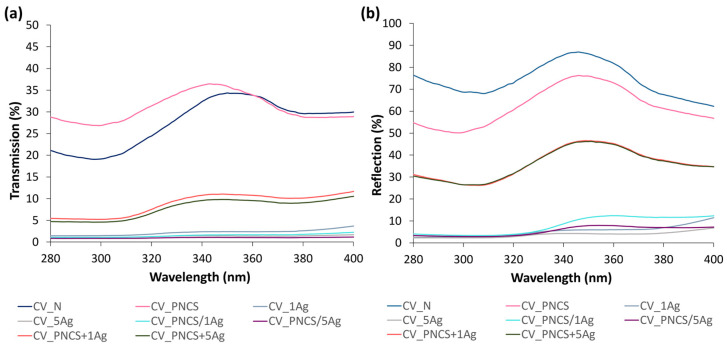
(**a**) UV transmission and (**b**) reflection spectra of untreated and studied modified samples.

The colouring of the modified samples by Ag NPs undoubtedly influenced the transmission properties and, thus, the UPF values determined; the presence of aromatic phenolic compounds in the sumac leaf extract, which act as UV absorbers, must also be taken into account. Accordingly, as can be seen in Figure 6, the in situ reduction in Ag^+^ by the sumac leaf extract (samples CV_xAg) resulted in excellent UV protection in the viscose fabric, as the transmittance decreased significantly, leading to higher UPF values (Table 2). This effect was even more pronounced in the CV_PNCS/xAg samples modified with functionalised PNCS hydrogel with Ag NPs synthesised in situ. While the CV_1Ag and CV_PNCS/1Ag samples, on the one hand, and the CV_5Ag and CV_PNCS/5Ag samples, on the other hand, contained a comparable amount of Ag, these results confirm that the PNCS hydrogel must have caused a higher uptake of the sumac leaf extract and, thus, a higher concentration of phenolic compounds, which further enhanced the UV-protective activity of the CV_PNCS/xAg samples; they exhibited a UPF of 76.46 and 73.39 compared to a UPF of 58.49 and 57.16 for the CV_1Ag and CV_5Ag samples, respectively. In contrast, the samples modified with the pre-functionalised PNCS hydrogel (CV_PNCS + xAg samples) exhibited a higher transmission and, consequently, lower UPF values, which did not provide satisfactory UV protection. These results were expected and are in good agreement with the values of the CIE L*a*b* colour co-ordinates (Figure 3a), as both CV_PNCS + xAg samples showed a light, slightly pale-yellow colour after application, which is in contrast to an intense brown colour of the CV_PNCS/xAg samples. Thus, the concentration of Ag NPs and phenolic components in the pre-functionalised PNCS hydrogel was too low to achieve a satisfactory UV protection in the CV_PNCS + xAg samples.

## 4. Conclusions

The results of this study demonstrate that PNCS hydrogel is a highly effective carrier for the in situ biosynthesis and stabilisation of Ag NPs on viscose fibres, highlighting in particular the crucial role of the application method. When the PNCS hydrogel was applied to viscose fibres and subsequently functionalized in situ, biosynthesised Ag NPs with exceptionally small sizes, which corresponds to the SPR peak at 388 nm, and of high concentrations, were formed on the fibre surface, which coloured the samples from brown to brown-green. This application process configuration granted a complementary effect between the PNCS hydrogel and the Ag NPs, showing certain advantages over pre-functionalised PNCS hydrogels. Accordingly, it resulted in excellent antibacterial activity, whereas the observed antibacterial properties (>90% efficacy against Gram-positive and Gram-negative bacteria) demonstrate the ability of the PNCS hydrogel as a reactive reservoir for Ag NPs. Below the lower critical solution temperature (LCST) of poly-NiPAAm, the PNCS hydrogel was in a swollen state, which allowed greater release of Ag NPs through its porous structure and created a larger antibacterial inhibition zone. Above the LCST, the hydrogel collapsed, limiting release to the Ag NPs at the fibre surface and creating a smaller zone of inhibition. This selective response behaviour, which follows a diffusion-controlled mechanism, shows a controllable, temperature-dependent antibacterial effect. In addition, the in situ functionalisation of the PNCS hydrogel also resulted in excellent UV protection (UPF 50+).

Although the Ag NPs reduced the temperature and pH responsiveness of the PNCS hydrogel, the responsiveness of the modified CV samples was significantly improved compared to the unmodified CV sample. It can be concluded that PNCS hydrogel is a versatile and adaptable carrier for Ag NPs that enables their controlled and sustained release, which could improve functionality of smart textiles in protective and antibacterial applications.

## Figures and Tables

**Table 1:** Sample codes with details of the corresponding application process.

Sample Code	Application Process	Details
CV_N	/	Unfinished
CV_xAg	In situ	The application of the polysiloxane matrix with the subsequent in situ synthesis of Ag NPs using the AgNO_3_ solution (x stands for the concentration of the AgNO_3_; i.e., x = 1.0 or 5.0 mM)
CV_PNCS	Pad-dry	The application of polysiloxane and PNCS hydrogel
CV_PNCS/xAg	Pad-dry + in situ	The application of the polysiloxane matrix, and PNCS hydrogel, and the in situ synthesis of Ag NPs using the AgNO_3_ solution (x stands for the concentration of the AgNO_3_; i.e., x = 1.0 or 5.0 mM)
CV_PNCS + xAg	Pad-dry	The application of the polysiloxane matrix and pre-functionalised PNCS hydrogel with Ag NPs synthesised using AgNO_3_ (x stands for the concentration of the AgNO_3_; i.e., x = 1.0 or 5.0 mM)

**Table 2:** UVB and UVA blocking activity of the untreated and studied modified samples with mean UPF values and protection categories determined by the Australian/New Zealand Standard Sun protective clothing—Evaluation and classification.

Sample	UVB Blocking (%)	UVA Blocking (%)	UPF	Protection Category ^1^
CV_N	57.75	62.54	2.43	I
CV_PNCS	53.92	60.44	2.25	I
CV_1Ag	98.45	97.47	58.49	E
CV_5Ag	98.33	97.91	57.16	E
CV_PNCS/1Ag	98.76	98.29	76.46	E
CV_PNCS/5Ag	98.68	98.49	73.39	E
CV_PNCS + 1Ag	86.22	80.99	6.83	I
CV_PNCS + 5Ag	87.30	84.14	7.54	I

^1^ I—insufficient; E—excellent.

## Data Availability

No new data were created or analysed in this study. Data sharing is not applicable to this article.
